# Voltage imaging as a window into neural computation

**DOI:** 10.1117/1.NPh.13.S2.S23202

**Published:** 2026-02-09

**Authors:** Joram Keijser, Sadra Sadeh

**Affiliations:** a University College London, UCL Queen Square Institute of Neurology, London, United Kingdom; b King’s College London, Centre for Developmental Neurobiology, London, United Kingdom; c The Francis Crick Institute, London, United Kingdom

**Keywords:** voltage imaging, neuroscience, neural computation

## Abstract

Neural computation spans a range of scales, from dendritic integration within individual neurons to collective dynamics across networks. Voltage imaging provides a powerful approach to study these processes, as it captures both spiking and subthreshold activity of genetically defined populations with high temporal resolution. We discuss the potential of voltage imaging to reveal how cellular properties such as neuronal input–output functions shape network dynamics and population manifolds. By bridging cellular mechanisms and system-level function, voltage imaging opens new opportunities to test multiscale theories of neural computation.

## 1 Introduction

Neural computation is fundamentally multiscale, spanning dendritic integration within individual neurons, synaptic plasticity across local circuits, and emergent dynamics in large networks. Understanding how these scales interact (e.g., how cellular mechanisms give rise to population-level function) requires experimental methods that capture the full spectrum of neural activity. This spectrum includes not only the suprathreshold spikes that drive neural transmission but also the subthreshold membrane potentials that underlie these spikes. Established methods face inherent limitations in bridging these scales. Patch-clamp recordings capture subthreshold activity but only from a few cells,^1^ limiting population-level insights. Extracellular electrodes detect spikes across many neurons but miss the subthreshold processes that shape network dynamics.^2^ Calcium imaging provides population-scale measurements but offers only a slow, indirect measure of spiking activity, obscuring both subthreshold dynamics and individual spikes.^3^

**Fig. 1:**
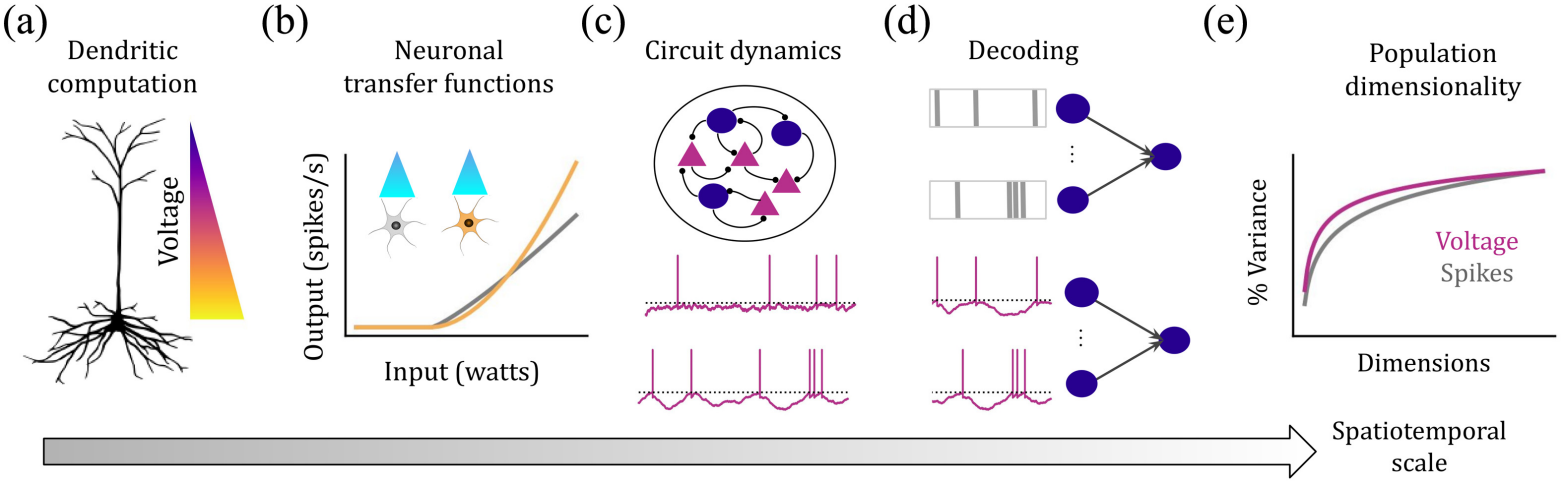
Voltage imaging as a window into neural computation across scales. Voltage imaging could **(a)** reveal dendritic computations by measuring voltage across entire dendritic trees, **(b)** probe cell-type-specific transfer functions using targeted optogenetics, **(c)** characterize the dynamical state of cortical circuits (e.g., mean-driven versus fluctuation-driven), **(d)** quantify the amount of information in subthreshold vs. suprathreshold activity, and **(e)** determine how spiking nonlinearities and sparsity shape the dimensionality of population activity.

The emerging method of voltage imaging uniquely addresses these limitations by directly measuring membrane potentials across genetically defined neural populations.^4^^–^^6^ This combination of single-cell resolution with population-scale coverage, coupled with millisecond temporal precision, positions voltage imaging as a powerful bridge between cellular and systems neuroscience. Here, we examine how voltage imaging’s unique capabilities can shed light on neural computation across organizational scales (Fig. 1). We begin by exploring single-neuron computations: how dendritic trees integrate synaptic inputs and how neurons transform these inputs into outputs. We then consider circuit-level phenomena, examining how synaptic connectivity evolves over time and how recurrent dynamics shape cortical states. Finally, we address population-level questions, investigating how cellular nonlinearities and spiking sparsity influence high-dimensional neural codes and whether subthreshold activity contains information hidden from traditional spike-based analyses.

**Fig. 2:**
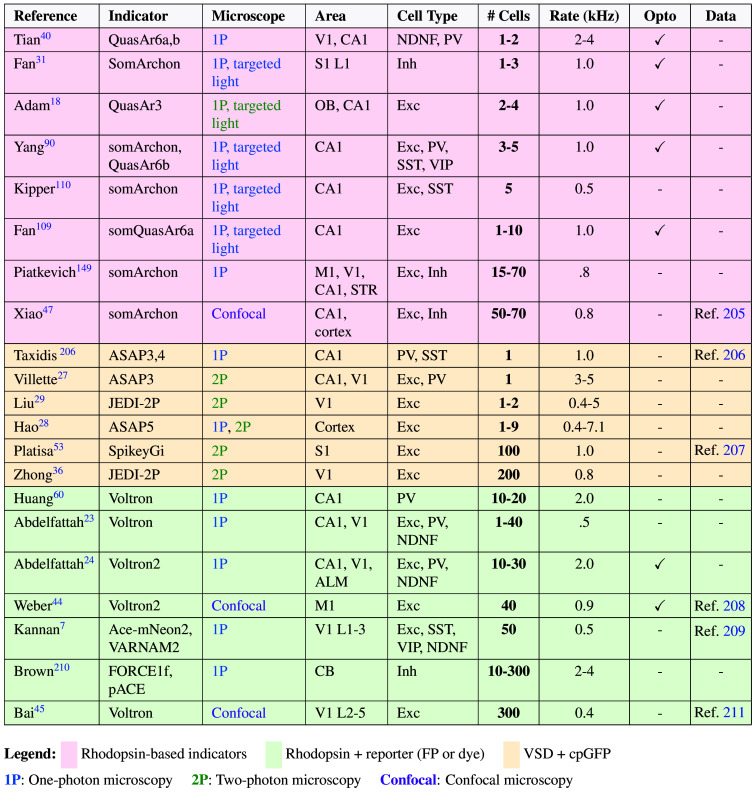
Examples of recent *in vivo* voltage imaging experiments, sorted by indicator and recording scale. Numerical values indicate reported experimental conditions rather than theoretical performance limits. Only recordings with single-cell resolution in mice are listed; recordings from other species are excluded for comparison. Cortical recordings were performed in layers 2 and 3, unless specified otherwise. **# Cells**: typical number of simultaneously recorded, spiking neurons (exact number varies between experiments and is mostly determined by microscopy parameters). **Rate**: scan rate in kilohertz, rounded to 1 decimal place. **Opto**: simultaneous voltage imaging and optogenetics. **Data**: reference to processed or raw data, if published. **Abbreviations**: FP, fluorescent protein; cpGFP, circularly permuted green fluorescent protein; VSD, voltage-sensing domain; L, cortical layer.

This multiscale perspective reflects voltage imaging’s potential to test theoretical frameworks that span organizational levels: from models of dendritic computation to theories of population coding. Because realizing this potential requires understanding current capabilities and limitations, we begin with a brief methodological introduction. Figure 2 provides an overview of recent studies to showcase what is achievable with existing technology.

### 1.1 Brief Introduction to Voltage Imaging

#### 1.1.1 Voltage indicators convert membrane potentials into fluorescence

Voltage imaging relies on genetically encoded voltage indicators (GEVIs) that convert membrane potentials into fluorescence. Because this fluorescence is a relative signal, voltage indicators do not measure absolute membrane voltage. To achieve cell-type-specific voltage measurements, several targeting strategies have been developed that restrict GEVI expression to particular neuronal populations. The simplest exploits the genetic encoding of GEVIs: injecting Cre-dependent viral vectors into Cre-driver lines restricts expression to a cell type of choice. For simultaneous monitoring, dual-color or dual-polarity imaging enables imaging of multiple cell types at once.^7^ Even more detailed cell-type information could be gleaned by combining *in vivo* imaging with *ex vivo* molecular identification.^8^^–^^15^

GEVIs employ diverse molecular designs, with prominent examples including (1) microbial rhodopsins performing both voltage sensing and fluorescence signaling;^16^^–^^18^ (2) hybrid designs coupling a voltage-sensing rhodopsin to a separate, brighter fluorescent reporter;^19^^–^^24^ and (3) hybrid designs coupling a voltage-sensing domain to a circularly permuted fluorescent protein (cpGFP).^25^^–^^29^ These designs have their own strengths and weaknesses; for example, the red-shifted spectrum of rhodopsin-based GEVIs (categories (1) and (2)) makes them compatible with blue-shifted optogenetic actuators,^18^^,^^30^^–^^32^ but they natively show limited voltage-sensitivity under two-photon excitation^23^^,^^26^^,^^33^^,^^34^ (although see Ref. 35). Similarly, many indicators are optimized for spike detection, motivating the development of complementary indicators targeting subthreshold activity.^28^

Because GEVIs reside in the cell membrane, they provide far fewer fluorophores per unit volume compared with calcium indicators. Together with the fast dynamics of voltage signals, this leads to lower signal-to-noise ratios (SNRs), necessitating higher irradiance for detection. (For example, Ref. 36 performed voltage and calcium imaging using the same microscope. Voltage imaging used 163 mW [post 1.05-NA objective] across a 160×400  μm plane, corresponding to an irradiance of $2.55\;W/{\mathrm{mm}}^{2}$. By contrast, calcium imaging used 102 mW [post 0.8-NA objective] across a 1111×1000×400  μm volume, corresponding to a surface irradiance of $∼0.09\;W/{\mathrm{mm}}^{2}$, i.e., 28-fold lower). This high illumination, in turn, causes photobleaching that typically limits recording durations to several minutes.

#### 1.1.2 Different microscopy systems can be used to detect the fluorescence

Detecting rapid, small-amplitude fluorescence changes requires microscopes with high illumination power and kilohertz imaging rates. The scale of this challenge is clear from comparison to calcium imaging: calcium events persist for ∼100  ms and induce fluorescence changes ($\Delta F/F_{0}$) on the order of 100%.^37^^–^^39^ By contrast, action potentials last ∼1  ms and produce $\Delta F/F_{0}$ on the order of 10%,^23^^,^^27^^,^^29^ with subthreshold voltage changes yielding even smaller signals.

The need for rapid imaging has traditionally precluded point-scanning approaches from two-photon microscopy, leading many voltage imaging experiments to use one-photon microscopy instead (Fig. 2). However, this approach suffers from increased background fluorescence because scattered light excites fluorophores outside the focal plane. Experimental strategies to minimize scattering and its effects include soma-localized, sparse labeling,^18^^,^^21^^,^^23^^,^^24^^,^^40^ and improved signal extraction software.^41^^–^^43^ Despite these approaches, single-photon voltage imaging has been limited to small populations in superficial layers. Recent microscopy advances that address these limitations include confocal scanning,^44^^,^^45^ targeted illumination,^31^^,^^46^ or a combination of the two.^47^ For transparent organisms such as zebrafish larvae, light sheet microscopy is an alternative.^23^^,^^48^^–^^51^ Other strategies adapt two-photon microscopy through spatiotemporal multiplexing,^36^^,^^52^^,^^53^ acousto-optic holography,^27^ or scanless imaging.^54^ The first commercially available two-photon microscopes capable of voltage imaging are now in use (e.g., Refs. 28 and 55). The choice of imaging parameters involves trade-offs, as imaging more cells typically increases the probability of missing spikes through reduced temporal resolution and SNR. Similarly, two-photon excitation allows imaging at greater depths than one-photon excitation, but requires higher laser powers.^56^ Different microscopy systems are therefore suitable for tackling different questions. Such trade-offs underscore the synergy between advances in voltage indicators and microscopy: newer indicators offer higher SNRs, thereby reducing the required laser intensity and enabling longer recordings at larger scales.

Although we focus primarily on imaging techniques with single-cell resolution, large-scale voltage dynamics can also be detected using widefield imaging.^57^^,^^58^ Although it lacks single-cell resolution, this approach can detect rapid, subthreshold activity invisible to calcium imaging. Together with its large spatial field of view, this enables, for example, the study of neural oscillations^59^^,^^60^ and traveling waves.^61^^,^^62^ Importantly, the weak illumination required allows imaging durations far longer than those possible with current cellular-resolution methods.^62^

#### 1.1.3 Processing voltage imaging data requires multiple algorithmic steps

Converting raw fluorescence to interpretable neural activity requires several processing steps^41^^,^^42^^,^^63^ (see Ref. 64 for a recent comparison). First, motion artifacts are corrected through image registration, typically using phase correlation methods to estimate temporal shifts.^65^ Second, cells and other regions of interest must be segmented from the background. Although manual segmentation remains common, semi-automatic approaches using matrix factorization^42^ or neural networks^66^ will become necessary as datasets increase in scale. Third, fluorescence signals are denoised and searched for spikes. Denoising can be done using smoothing, low-pass filtering, or self-supervised machine learning.^53^^,^^67^ Spike detection typically uses unsupervised methods based on adaptive thresholding or iteratively learned templates.^23^^,^^41^ Beyond spike detection, extracting subthreshold signals poses an additional challenge given their low amplitude and susceptibility to motion and hemodynamic artifacts.^68^ A rigorous approach to remove these contaminating signals uses the concurrent measurement of a voltage-independent fluorophore as a reference channel, as has been demonstrated for widefield imaging^62^^,^^69^

Although these steps parallel calcium imaging pipelines,^70^^–^^72^ voltage imaging presents distinct challenges, such as high acquisition rates, fast photobleaching, and low SNR. Addressing these challenges will likely require publicly available datasets and standardized benchmarks. For example, simultaneous voltage imaging and electrophysiology would enable the development and comparison of spike detection methods (cf. Refs. 73 and 74).

#### 1.1.4 Summary of technical challenges

In sum, voltage imaging requires careful integration of indicators, microscopes, and algorithms. Although substantial progress has been made in each area, fundamental trade-offs remain. Strong illumination causes photobleaching, which limits recording durations, whereas the high imaging speeds required to capture fast voltage dynamics limit the number of cells that can be recorded simultaneously. In addition, motion and hemodynamic artifacts complicate the extraction of subthreshold signals. Consequently, no single combination of indicator and imaging modality suits all spatiotemporal scales, requiring researchers to match their technical approach to their scientific questions.

## 2 How do Dendrites Contribute to Neural Computation?

Neurons possess elaborate dendritic trees that support active, nonlinear signal processing.^75^ Foundational insights into these properties have come from painstaking patch-clamp recordings revealing how dendritic and somatic activity interact.^76^^,^^77^ More recently, this question has been addressed using quasi-simultaneous calcium imaging of somata and dendrites.^78^^,^^79^ Although important, this approach cannot capture subthreshold fluctuations such as hyperpolarization through which inhibition may gate inputs and synaptic plasticity.^80^^–^^82^

Voltage imaging experiments can build on this foundational work by enabling measurements across the entire dendritic tree, rather than at the few discrete sites accessible to electrodes. This capability is especially powerful when combined with optogenetic stimulation, as illustrated by a recent all-optical approach for probing CA1 pyramidal cells.^32^ By pairing a red-shifted GEVI with a blue-shifted channelrhodopsin,^17^ the authors replicated classic patch-clamp findings such as the dependence of dendritic spikes on prior depolarization.^76^ Simultaneous voltage imaging across dendrites further revealed a narrow temporal window for dendritic spike initiation, consistent with their role in coincidence detection.^76^ This work also revealed a lack of branch-level compartmentalization, potentially limiting the computational capacity of individual dendritic branches. By contrast, other voltage imaging experiments have reported compartmentalization at the finer scale of dendritic spines.^83^

Once possible, analogous *in vivo* experiments could contribute to our understanding of behaviorally related dendritic computation. In particular, this could determine whether apical dendrites act as integration sites for top-down and bottom-up inputs.^84^^–^^86^ In theories of predictive coding, for example, apical dendrites are often considered the recipients of predicted sensory inputs as well as inhibitory inputs that can balance these predictions.^84^^,^^87^^–^^89^ Voltage imaging in mice that learn to expect particular stimuli based on their sensory or spatial contingencies could reveal whether dendrites (but not somata) indeed receive predictive inputs, and whether these predictions are cancelled by particular inhibitory cells.

Finally, voltage imaging can distinguish somatic spikes from dendritic bursts,^90^ which may carry distinct signals.^85^^,^^91^ Recent experiments suggest, for example, that the apical dendrites of CA1 pyramidal cells might receive an instructive signal for plasticity induction.^92^ Theoretical work has suggested that the separation of somatic from dendritic activity could enable biological approximations of the backpropagation algorithm.^93^^–^^96^ Voltage imaging could determine whether dendrites indeed receive the required multi-dimensional error or target signals. Particularly useful for studying learning will be the ability, enabled by imaging experiments, to track neurons and dendrites across task acquisition.^97^^–^^99^

Together, these directions suggest that voltage imaging will play an important role in the study of dendritic computation. Realizing this potential will require solving technical challenges to achieve *in vivo* imaging of subthreshold signals throughout individual neurons.

## 3 How do Synaptic Connectivity and Neural Representations Change Over Time?

The ability to measure both subthreshold and spiking activity makes voltage imaging a powerful tool for studying synaptic plasticity. Although spike timing and other suprathreshold events have long dominated theories of synaptic plasticity,^100^^–^^102^ subthreshold activity can also shape these processes.^103^^–^^107^ Optical recordings could measure this voltage dependence on a larger scale than patch-clamp recordings. For example, behavioral timescale synaptic plasticity (BTSP) produces seconds-long ramping depolarisation in CA1 pyramidal neurons through an asymmetric plasticity kernel.^108^ Voltage imaging experiments have shown that BTSP requires presynaptic activity in CA2/3^109^ and have also revealed a more symmetric depolarization ramp in somatostatin-positive neurons, suggesting they follow a cell-type-specific plasticity rule.^110^ Not all neural changes coincide with overt learning or adaptation. In some cases, the correlations between neural responses and sensory input or spatial location gradually change over days to weeks without clear accompanying changes in behavior.^111^^–^^113^ The causes and consequences of these gradual changes, known as representational drift, are currently unclear.^114^^–^^116^ They could be studied using voltage imaging, as the spatial resolution of optical recordings enables longitudinal tracking of individual cells.^110^^,^^117^ Although this is now also possible with electrophysiological recordings,^118^^,^^119^ voltage imaging also measures subthreshold dynamics. This could, for example, reveal whether unreliable spiking reflects sparse firing atop stable inputs or actual changes in synaptic drive. Careful behavioral monitoring will be essential to further distinguish genuine drift from variability introduced by changes in behavioral state.^120^^–^^125^

In sum, by combining subthreshold measurements with longitudinal single-neuron tracking, voltage imaging offers unique opportunities to dissect the mechanisms underlying both synaptic plasticity and representational drift.

## 4 How do Neurons Transform their Inputs into Outputs?

The shape of a neuron’s input–output (I/O) function (namely, how it translates synaptic input into spiking activity) has important consequences for neural computation and network dynamics. For example, a power-law I/O function combined with stabilizing inhibition may account for multiple nonlinear response properties of the visual cortex.^126^^–^^129^ An expansive nonlinearity is consistent with the power-law dependence of firing rate on membrane potential observed through whole-cell recordings from excitatory neurons in anesthetized cats^130^^,^^131^ and mice.^132^^,^^133^ However, I/O functions likely vary between cell types and behavioral states,^134^ and critically, a power-law voltage-rate relationship need not imply that the I/O function itself is a power-law: at higher rates, spike-frequency adaptation and refractory periods may flatten the relationship between inputs and outputs^135^—something that voltage-rate curves fail to capture.

Voltage imaging is uniquely suited to address these issues. Recent work used voltage imaging to replicate the power-law relationship between voltage and spike rate, now in hundreds of pyramidal cells in awake mice.^36^ Crucially, the compatibility of voltage imaging with cellular-resolution optogenetics enables the direct, causal probing of I/O functions, which has already revealed the state-dependence of I/O functions in behaving mice.^18^^,^^90^^,^^109^ Future experiments will need to test the effects of optogenetic stimulation across a range of physiological activity levels, because sufficiently strong inputs will inevitably lead to saturation, potentially masking the true I/O shape. A recent calcium imaging experiment achieved this by comparing the effects of optogenetic stimulation during spontaneous versus evoked activity.^136^ Instead of a power-law I/O function, this revealed proportional rather than amplified outputs above baseline activity levels.^136^ Analogous voltage imaging experiments could unambiguously identify baseline activity in terms of firing rates, overcoming potential limitations of calcium-based spike inference.

Voltage imaging can therefore reveal the contribution of I/O functions to neural computation through large-scale measurements across different cell types.

## 5 What Mechanisms Shape Cortical Circuit Dynamics?

Cortical networks can occupy a wide range of multi-dimensional states, from synchronized, oscillatory activity to asynchronous dynamics.^122^^,^^137^^–^^139^ This spontaneous activity is thought to reflect internal states such as arousal or attention and can influence information processing.^137^^,^^140^^,^^141^ Researchers typically characterize these dynamics through pairwise correlations,^142^^–^^144^ oscillatory power,^121^^,^^138^ and population geometry.^145^^–^^148^ However, such analyses have primarily relied on spiking activity or calcium concentration, which provide only a partial view of network dynamics. Voltage imaging, by contrast, can also measure subthreshold activity underlying population activity, potentially revealing how synaptic integration and intrinsic cellular properties generate network-level patterns. Indeed, voltage imaging has been used to measure state-dependent pairwise correlations between subthreshold fluctuations of pyramidal cells^18^ and spiking activity of diverse interneurons.^7^ Voltage imaging has also confirmed the stronger coupling of CA1 cells to their own intracellular theta-rhythm than to that in the local field potential.^149^

Earlier theoretical work has shown how network states such as synchronization are shaped by the balance of excitation and inhibition.^150^^–^^153^ Although experiments confirm that excitation and inhibition are indeed balanced,^154^^–^^158^ recordings also reveal discrepancies with these models: non-zero pairwise correlations^143^ and multi-dimensional population activity^122^^,^^139^ that contrast with vanishingly small correlations and lack of shared activity patterns predicted by theory.^150^^–^^152^^,^^159^^,^^160^

This mismatch could arise from several factors absent from classical models, including structured recurrent connectivity,^161^^–^^164^ correlated external inputs,^165^^–^^167^ and neuromodulation.^14^^,^^168^^,^^169^ Subthreshold measurements are crucial to distinguish cortical states and the underlying mechanisms, whereas fluctuation-driven asynchronous states are associated with Gaussian-distributed membrane potentials, synchronous states are associated with skewed distributions away from threshold.^120^^,^^151^^,^^170^ Voltage imaging can build on foundational results from patch-clamp recordings by simultaneously recording subthreshold activity across many neurons to characterize the mechanisms underlying shared population activity.

Combining voltage imaging with cellular-resolution optogenetics^171^ could illuminate the role of recurrent connectivity. This approach has already revealed an important role of recurrence in shaping sensory responses when combined with calcium imaging.^172^^,^^173^ Analogous optopatch experiments using voltage imaging^17^^,^^18^^,^^174^ would exploit its increased temporal resolution to disentangle direct synaptic connections from higher-order network effects. This approach has already revealed electrical coupling between PV-positive CA1 cells and confirmed the synaptic coupling between cortical NDNF-positive cells^40^ (cf. Ref. 175). Access to subthreshold signals would also uncover effects invisible in sparse firing patterns; for instance, whether silent neurons are genuinely unaffected or actually inhibited during a perturbation.

Voltage imaging can therefore contribute to our understanding of circuit dynamics by simultaneously and cell-type-specifically measuring subthreshold dynamics. Fully realizing this promise will require further advances in indicator photostability to enable sustained recordings *in vivo* recordings.

## 6 How do Neuronal Nonlinearities and Spiking Shape Population Activity?

Analyses of neural population activity often focus on its dimensionality, i.e., the number of independent encoded variables.^176^^,^^177^ Although dimensionality is typically interpreted in the context of population-level interactions, single-neuron properties also contribute; for example, a low-rank recurrent neural network^178^^,^^179^ can produce high-dimensional activity if its units have nonlinear transfer functions.^180^ Spiking-induced sparsity may further increase dimensionality by reducing correlations and redundancy.^181^^–^^183^ The effects of such single-neuron properties are currently unknown, as the study of population-level activity has been based on suprathreshold measurements.^145^^,^^146^^,^^148^^,^^184^

By measuring spiking and subthreshold activity, large-scale voltage imaging could clarify how single-cell nonlinearities and sparsity shape population activity. This will require mitigating challenges for population analyses of subthreshold recordings, such as motion artifacts^27^ and variable signal-to-noise levels across cells.^36^

Comparing the dimensionality of subthreshold versus spiking population activity should reveal whether the latter is higher-dimensional, as expected from its higher sparsity. Once experimentally feasible, voltage imaging across 1000s of cells could reveal how sparsity influences the smoothness of high-dimensional population codes.^185^

Beyond sparsity, the role of neuronal nonlinearities can be analyzed by modeling spiking activity as a linear-nonlinear combination of latent factors.^180^^,^^186^^,^^187^ An open question is whether such methods enable an accurate reconstruction of subthreshold fluctuations; although subthreshold fluctuations cannot reliably be inferred from the spiking activity of isolated cells,^188^^,^^189^ this inference could be feasible with access to (correlated) population activity.^190^

Voltage imaging could therefore reveal how population-level concepts, such as dimensionality, are shaped by interpretable single-neuron properties, such as transfer functions. Realizing this potential will require advances in high-speed microscopy to enable large-scale population recordings.

## 7 Do Subthreshold Potentials Contain Information Hidden in Spiking?

Sparse neural responses likely reflect efficient coding because action potentials account for a large fraction of the brain’s energy consumption,^191^ they should be kept to a minimum.^181^^,^^183^^,^^192^ Suprathreshold activity must therefore balance information transmission and metabolic costs. By contrast, subthreshold activity is metabolically cheaper and may support richer, less constrained representations.

Consider a neuron that receives strong input for preferred stimuli but weak, subthreshold input for non-preferred stimuli. Its spiking output will be largely uninformative about these non-preferred stimuli, even if they elicit distinct subthreshold membrane potentials. In this way, sparse spiking activity may miss information readily available at the subthreshold level.

Supporting this idea, a recent study revealed spatially tuned subthreshold inputs to non-place cells through optogenetic depolarization.^193^ Voltage imaging could extend this approach by quantifying the additional information contained in subthreshold versus suprathreshold activity. Practically, voltage imaging could improve the efficiency of brain–computer interfaces^194^ by requiring fewer neurons to achieve a given performance level. Conceptually, voltage imaging could reveal insights into circuit mechanisms—for example, by showing whether a neuron’s tuning arises from many broadly tuned inputs or a few sharply tuned inputs.

Such mechanistic insights would be particularly useful to explain the different coding schemes found across cell types. In the primary sensory cortex, for example, inhibitory cells are typically less stimulus-selective than excitatory cells.^8^^,^^195^^–^^197^ Paired with neurotransmitter imaging,^198^^–^^200^ voltage imaging could determine whether this difference reflects variation in connectivity, synaptic properties, or intrinsic excitability. Paired with computational modeling, voltage imaging could determine when spiking-induced sparsity and decorrelation actually improve neural information capacity at the population level.^201^^–^^203^

Beyond sensory coding, widefield imaging of voltage dynamics has already been used to study oscillations and travelling waves.^62^ It has also revealed state-dependent synchrony in CA1^30^ and locomotion-coupled delta oscillations in individual striatal cells.^204^ Subthreshold activity could thus reveal the full information encoded by neurons and uncover computations hidden from spiking output.

## 8 Conclusion

By capturing detailed single-neuron activity across increasingly large populations, voltage imaging enables investigation of neural computation across scales, from dendritic integration and input–output functions within single neurons to synaptic plasticity and circuit dynamics in local circuits, and ultimately to population coding and manifold structure in large networks. Here, we discussed how voltage imaging could illuminate neural computation across these levels of description (see Table 1 for a summary).

**Table 1:** Summary of key questions that could be answered using voltage imaging.

Topic	Questions
Dendritic computation	Do dendrites act as specialized sites for integrating top-down and bottom-up inputs?
	How do different interneuron types gate dendritic spikes?
Input–output functions	Do neuronal input–output functions follow a power-law?
	How do input–output functions vary across cell types and behavioral states?
Circuit dynamics	What explains the discrepancy between classical balanced network models (which predict near-zero correlations) and observed population activity patterns?
	Do cortical networks maintain excitation-inhibition balance through fast feedback inhibition, as proposed by stabilised (supralinear) network theory?
Plasticity and learning	Does representational drift reflect noise in synaptic weights or systematic reorganisation driven by ongoing learning algorithms?
	How do subthreshold voltage dependencies of plasticity contribute to the formation of structured connectivity?
Population manifolds	Does spiking nonlinearity increase the dimensionality of population codes compared to their subthreshold counterparts?
	How does sparsity affect the smoothness and geometry of high-dimensional neural manifolds?
Encoding and decoding	Do sparse firing patterns increase information capacity or do they reduce information by discarding subthreshold signals?
	Can efficient coding principles explain the relationship between subthreshold selectivity and spiking sparsity across different cell types?
Cross-scale integration	How do single-neuron nonlinearities propagate through recurrent networks to shape population-level dynamics?
	Can bottom-up models starting from detailed neuron models predict the macroscopic network states observed in voltage imaging data?

This potential stems from voltage imaging’s unique capabilities, unmatched by either electrophysiology or calcium imaging. It combines the high temporal resolution to capture fast subthreshold dynamics with the spatial scale to record from neural populations. Compatibility with optogenetics enables all-optical circuit dissection while genetically encoded indicators afford cell-type specificity. Moreover, its optical accessibility enables longitudinal tracking of individual neurons over extended time scales. To fully exploit these capabilities, current limitations—particularly recording duration and scale—must be addressed through continued improvements in voltage indicators, microscopy, and processing software.

By bridging cellular mechanisms with system-level function, voltage imaging will open new opportunities for developing and testing multiscale theories of neural computation.

## Data Availability

No new code, data, or other materials were generated in this study.
